# Possible limited justification for systematic head computed tomography scans based solely on antithrombotic therapy in elderly patients (aged 75 or older) with mild traumatic brain injury

**DOI:** 10.1016/j.redii.2024.100053

**Published:** 2025-01-16

**Authors:** Emma Jaffres, Jean-Nicolas Dacher, Mehdi Taalba, Frédéric Roca, Matthieu Garnier, Sébastien Normant, Mathieu Lozouet, Emmanuel Gérardin, Julien Burel

**Affiliations:** a Department of Radiology, Centre Hospitalier Universitaire de Rouen, Rouen, France; b Department of Emergency Medicine, Centre Hospitalier Universitaire de Rouen, Rouen, France; c Department of Geriatric Medicine, Centre Hospitalier Universitaire de Rouen, Rouen, France; d Department of Neurosurgery, Centre Hospitalier Universitaire de Rouen, Rouen, France

**Keywords:** Elderly patients, Computed tomography, Antithrombotics, Traumatic cerebral hemorrhage, Mild traumatic brain injury

## Abstract

**Rationale and objectives:**

Recent literature suggests that performing systematic head computed tomography (CT) scans for mild traumatic brain injury (mTBI) in patients undergoing antithrombotic therapy offers limited benefits. This study aims to evaluate a set of criteria that could potentially eliminate the need for systematic head CT scans, performed solely because of the antithrombotic treatment status, in elderly patients (aged 75 or older) presenting with mTBI.

**Materials and methods:**

All patients aged 75 or older who underwent a head CT scan at our academic center for mTBI while on antithrombotic therapy between January and December 2022 were retrospectively included in this study. Patients were categorized into two groups. The first group, referred to as the “At-risk group”, included patients with any of the following: GCS score < 15 or cognitive impairment; initial loss of consciousness; hemodynamic instability; signs of fractures; extensive subcutaneous hematoma; severe or treatment-resistant headache; vomiting; seizure; any neurological deficit; intoxication; amnesia; or a history of neurosurgery. The second group, referred to as the “Not-at-risk group”, comprised patients without any of these criteria.

**Results:**

A total of 1415 patients were included. Post-traumatic intracranial hemorrhage (*P* < 0.001), brain herniation (*P* = 0.003), and fractures (*P* < 0.001) occurred statistically more frequently in the At-risk group. Six post-traumatic hemorrhagic brain injuries were found in the Not-at-risk group, that did not present any of the studied criteria, and all these injuries were minor (localized SAH; millimetric SDH). Furthermore, none of these required immediate or delayed surgical intervention, and no neurological deterioration or deaths occurred in these patients.

**Conclusion:**

In conclusion, conducting systematic head CT scans based solely on antithrombotic therapy in elderly patients aged 75 or older with mTBI might be irrelevant.

**Abbreviations**
mTBI: mild traumatic brain injury
GCS: Glasgow coma scale
CT: computed tomography
mRS: modified Rankin scale
PACS: picture archiving and communication system
SD: standard deviation
IQR: interquartile range
SPSS: Statistical Package for the Social Sciences

## 1 Introduction

Mild traumatic brain injury (mTBI) refers to an acute brain injury resulting from the transfer of mechanical energy to the head by external physical forces, with a Glasgow Coma Scale (GCS) score following mTBI ranging from 13 to 15. It encompasses impacts to the head as well as acceleration and deceleration movements without direct trauma to the skull. It is estimated that globally, 90 % of all head injuries are mTBIs, making them a common reason for visits to emergency departments [[1], [2], [3], [4]].

The management of traumatic brain injury represents a significant socio-economic cost, amounting to nearly 400 billion dollars annually worldwide [5]. The transfer of a patient with mTBI to a hospital facility equipped with an emergency department, along with the use of computed tomography (CT) for diagnostic purposes, contributes to higher public health costs. A reduction in the number of CT scans performed in the aftermath of a traumatic brain injury would constitute a major medical-economic saving [6,7]. mTBIs disproportionately impacts the elderly, especially those over the age of 75, who represent a unique patient demographic. Within this population, injuries frequently result from low-kinetic events, most commonly falls from standing height [8]. Additionally, the options for surgical intervention are constrained for these patients [9]. Recognizing that moving an elderly person from their usual living environment can exacerbate neurological issues, the practice of transferring mTBI patients for routine emergency imaging is subject to debate [10]. This concern is particularly relevant for individuals living in institutional settings, such as retirement or nursing homes, where while supervision is provided, there may be an absence of on-site facilities capable of conducting brain CT scans.

Recent literature suggests that performing systematic head CT scans for mTBI in patients undergoing antithrombotic therapy offers limited benefits, particularly in adults younger than 65 years of age [1,3]. However, the necessity of such diagnostic procedures in elderly patients remains uncertain. This study aims to evaluate a set of criteria that could potentially eliminate the need for systematic head CT scans, performed solely because of the antithrombotic treatment status, in elderly patients (aged 75 or older) presenting with mTBI.

## 2 Materials and methods

### 2.1 Study design and patient characteristics

All patients who underwent a head CT scan at our academic center for mild traumatic brain injury while on antithrombotic therapy are included in a database. Patients who underwent head CT scan between January 2022 and December 2022 were retrospectively included in this study using this database.

The inclusion criteria were as follows: patients aged ≥ 75 years, treated with antithrombotics (either anticoagulants or antiplatelets), who presented at the emergency department within 24 h after trauma, with a GCS score of 13–15. The exclusion criteria were as follows: age < 75 years, GCS < 13, transfer from other hospitals, high-impact trauma (high kinetics).

Clinical characteristics of the patients were documented from both paper medical records and digital files. Information gathered included age, sex, location of the trauma (such as own residence, retirement home, nursing home, or hospital), modified Rankin scale (mRS) score, type of antithrombotic medication.

Patients were categorized into two groups, with criteria inspired by current guidelines from France, Canada, the United Kingdom, and the United States [1,[11], [12], [13]]. The first group, referred to as the “At-risk group”, included patients with any of the following, either alone or in combination: a GCS score < 15 or cognitive impairment; initial loss of consciousness; hemodynamic instability; signs of skull vault, skull base, or facial fractures (including otorrhagia, rinorrhagia, spectacle hematoma, etc.); extensive subcutaneous hematoma; severe or treatment-resistant headache; vomiting; seizure; any neurological deficit; drug or alcohol intoxication; amnesia related to the event; or a history of neurosurgery. The second group, referred to as the “Not-at-risk group”, comprised patients without any of these criteria.

Outside of the initial visit to the emergency department, which could occasionally be followed by hospitalization within the same institution, no systematic follow-up protocol was implemented. Consequently, data on patient outcomes beyond this period were not routinely collected.

### 2.2 CT scan protocol and image analysis

CT scans were conducted without the injection of contrast medium, utilizing volume spiral acquisition from the cranio-cervical junction to the vertex, and a section thickness of 1.25 mm. The studies from all patients were placed in an anonymized folder on the picture archiving and communication system (PACS). The CT scans were independently reviewed by a neuroradiology resident and a senior neuroradiologist. In cases of disagreement, a consensus was reached between the two radiologists.

The elements gathered included the presence or absence of intracranial hemorrhage; the type of hemorrhage, categorized into petechial hemorrhages, parenchymal hematoma, subarachnoid hemorrhage, extradural hematoma, subdural hematoma, and intraventricular hemorrhage; presence or absence of brain herniation; type of herniation; and the presence or absence of skull vault, skull base, or facial fractures, along with the type of fracture.

Due to the retrospective nature of this study and missing data, it was not possible to determine the precise average time interval between trauma and the completion of the CT scan.

### 2.3 Statistical analysis

Continuous data are presented as mean (standard deviation [SD]) or median (interquartile range [IQR]). Categorical variables are presented as count (percentage). Statistical comparisons were performed by a Student *t*-test for normally distributed data, the Mann-Whitney U test for data with a skewed distribution, and the ꭓ² and Fisher's exact tests for categorical data. A P value < 0.05 was statistically significant. The data were analyzed using the Statistical Package for the Social Sciences (SPSS; Version 28.0.1.1, IBM, Armonk).

## 3 Results

### 3.1 Patient characteristics

Table 1 presents the descriptive data for the study population, categorized according to the two groups previously defined.

**Table 1:** Study on the relevance of systematic head CT scans based solely on antithrombotic therapy in elderly patients aged 75 or older with mild traumatic brain injury: Descriptive data for the study population.

	Total (*n* = 1415)	At-risk group (*n* = 772, 54.6 %)	Not-at-risk group (*n* = 643, 45.4 %)	*P*
Age (years), median (IQR)	87 (82–91)	87 (82–91)	87 (82–91)	0.613
Women, count (percentage)	833 (58.9 %)	459	374	0.623
Location of the trauma, count (percentage)				
- Own residence	999 (70.6 %)	529 (68.5 %)	470 (73.1 %)	0.06
- Retirement home	84 (5.9 %)	43 (5.6 %)	41 (6.4 %)	0.523
- Nursing home	211 (14.9 %)	143 (18.5 %)	68 (10.6 %)	**< 0.001**
- Hospital	121 (8.6 %)	57 (7.4 %)	64 (10.0 %)	0.085
Initial mRS, median (IQR)	3 (3–3)	3 (3–3)	3 (3–3)	**< 0.001**
Antiplatelet therapy, count (percentage)	729 (51.5 %)	398 (51.6 %)	331 (51.5 %)	0.977
Anticoagulant therapy, count (percentage)	722 (51.0 %)	390 (50.5 %)	332 (51.6 %)	0.676
Other hemostasis issue, count (percentage)	7 (0.5 %)	1 (0.1 %)	6 (0.9 %)	0.051
**Criteria related to the trauma (and assessed in the present study)**, count (percentage)				
- GCS < 15 or cognitive impairment	474 (33.5 %)	474 (61.4 %)	0 (0.0 %)	NA
- Initial loss of consciousness	89 (6.3 %)	89 (11.5 %)	0 (0.0 %)	NA
- Hemodynamic instability	6 (0.4 %)	6 (0.8 %)	0 (0.0 %)	NA
- Sign of fracture	100 (7.1 %)	100 (13.0 %)	0 (0.0 %)	NA
- Extensive subcutaneous hematoma	175 (12.4 %)	175 (22.7 %)	0 (0.0 %)	NA
- Severe or treatment-resistant headache	29 (2.0 %)	29 (3.8 %)	0 (0.0 %)	NA
- Vomiting	26 (1.8 %)	26 (3.4 %)	0 (0.0 %)	NA
- Seizure	8 (0.6 %)	8 (1.0 %)	0 (0.0 %)	NA
- Neurological deficit	15 (1.1 %)	15 (1.9 %)	0 (0.0 %)	NA
- Drug or alcohol intoxication	16 (1.1 %)	16 (2.1 %)	0 (0.0 %)	NA
- Amnesia related to the event	92 (6.5 %)	92 (11.9 %)	0 (0.0 %)	NA
- History of neurosurgery	23 (1.6 %)	23 (3.0 %)	0 (0.0 %)	NA

At-risk group: patients meeting any of the criteria assessed; Not-at-risk group: patients meeting none of the criteria; IQR: interqurtile range; mRS: modified Rankin scale; GCS: Glasgow coma scale.

In the At-risk group, 390 (50.5 %) were treated with anticoagulant therapy, which included 315 (40.8 %) on direct oral anticoagulants (DOACs), 63 (8.2 %) on vitamin K antagonists (VKAs), 12 (1.6 %) on low-molecular-weight heparin (LMWH), and 0 (0.0 %) on unfractionated heparin (UFH). Additionally, 398 (51.6 %) patients of this group were treated with antiplatelet therapies: 332 (43.0 %) on aspirin, 51 (6.6 %) on clopidogrel, 0 (0.0 %) on prasugrel, 0 (0.0 %) on ticagrelor, and 15 (1.9 %) undergoing dual antiplatelet therapy. Notably, 1 (0.1 %) of this group had other hemostasis issues, such as advanced cirrhosis.

In the Not-at-risk group, 333 (51.8 %) were treated with anticoagulant therapy, which included 264 (41.1 %) on DOACs, 53 (8.2 %) on VKAs, 14 (2.2 %) on LMWH, and 2 (0.3 %) on UFH. 331 (51.5 %) patients of this group were treated with antiplatelet treatments: 266 (41.4 %) on aspirin, 45 (7.0 %) on clopidogrel, 0 (0.0 %) on prasugrel, 0 (0.0 %) on ticagrelor, and 20 (3.1 %) undergoing double antiplatelet therapy. 6 (0.9 %) of this group had other hemostasis issues.

### 3.2 Traumatic injuries

Table 2 delineates the types of traumatic injuries identified in the two groups previously mentioned. It categorizes intracranial hemorrhages into petechial hemorrhages, parenchymal hematomas, subarachnoid hemorrhages, extradural hematomas, subdural hematomas, and intraventricular hemorrhages. Additionally, the table classifies brain herniations, detailing subfalcine herniation, central herniation, uncal herniation, and tonsillar herniation.

**Table 2:** Study on the relevance of systematic head CT scans based solely on antithrombotic therapy in elderly patients aged 75 or older with mild traumatic brain injury: Traumatic injuries identified in the two groups studied.

	Total (*n* = 1415)	At-risk group (*n* = 772, 54.6 %)	Not-At-risk group (*n* = 643, 45.4 %)	*P*
**Intracranial hemorrhages (overall)**	88 (6.2 %)	82 (10.6 %)	6 (0.9 %)	**< 0.001**
- Petechial hemorrhages	16 (1.1 %)	16 (2.1 %)	0 (0.0 %)	
- Parenchymal hematomas	11 (0.8 %)	11 (1.4 %)	0 (0.0 %)	
- Subarachnoid hemorrhages	34 (2.4 %)	30 (3.9 %)	4 (6.2 %)	
- Extradural hematomas	1 (0.1 %)	1 (0.1 %)	0 (0.0 %)	
- Subdural hematomas	44 (3.1 %)	43 (5.6 %)	1 (0.2 %)	
- Intraventricular hemorrhages	5 (0.4 %)	4 (0.5 %)	1 (0.2 %)	
**Brain herniations (overall)**	10 (0.7 %)	10 (1.3 %)	0 (0.0 %)	**0.003**
- Subfalcine herniation	7 (0.5 %)	7 (0.9 %)	0 (0.0 %)	
- Central herniation	0 (0.0 %)	0 (0.0 %)	0 (0.0 %)	
- Uncal herniation	3 (0.2 %)	3 (0.4 %)	0 (0.0 %)	
- Tonsillar herniation	0 (0.0 %)	0 (0.0 %)	0 (0.0 %)	
**Fractures (overall)**	80 (5.7 %)	73 (9.5 %)	7 (10.9 %)	**< 0.001**

**Fig. 1:**
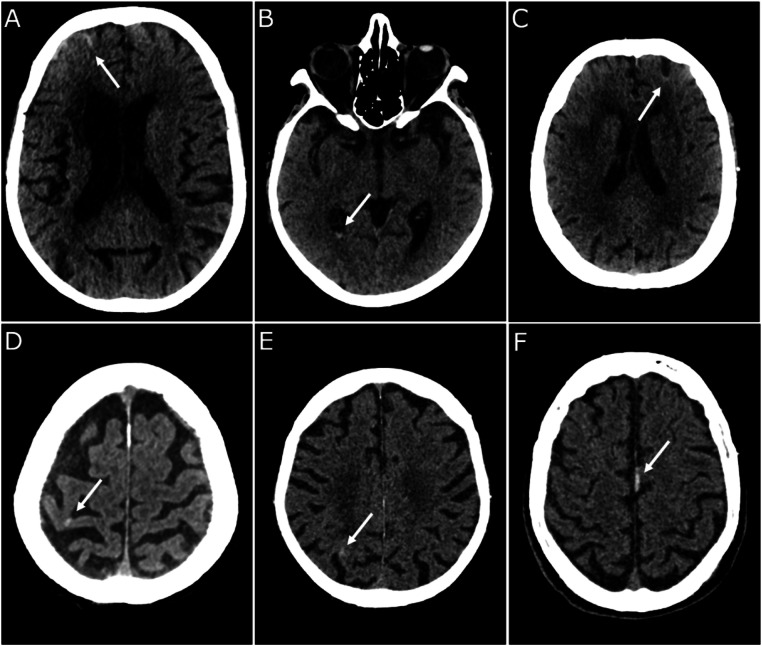
Study on the relevance of systematic head CT scans based solely on antithrombotic therapy in elderly patients aged 75 or older with mild traumatic brain injury: Brain CT scans from the six patients in the Not-at-risk group who experienced intracranial hemorrhage. White arrows are used to indicate the locations of the intracranial hemorrhages. Patients 1, 3, 4, and 5, corresponding to panels A, C, D, and E, experienced subarachnoid hemorrhages. Patient 2, corresponding to panel B, experienced intraventricular hemorrhage. Patient 6, corresponding to panel F, experienced subdural hematoma. All of these cases corresponded to minor hemorrhages, and no instances of brain herniation were detected.

In the Not-at-risk group, six patients (0.9 %) were found to have an intracranial hemorrhage. All of these cases corresponded to minor hemorrhages, and no instances of brain herniation were detected in this group. Fig. 1 depicts the six cases of intracranial hemorrhage in this group.

In the At-risk group, 73 (9.5 %) fractures were identified, with 32 (4.1 %) consisting of nasal fractures, 13 (1.7%) consisting of fractures of the orbital floor, 10 (1.3 %) consisting of fractures of other walls of the maxillary sinus, 6 (0.8 %) consisting of fractures of the atlantoaxial complex, 6 (0.8 %) consisting of not depressed skull fractures, 3 (0.4 %) were LeFort type 2 or 3 fractures, 2 (0.3 %) consisting of fractures of the orbital roof, and 1 (0.1 %) consisting of fractures of the petrous part of the temporal bone.

In the Not-at-risk group, 7 (1.1 %) fractures were identified, with 2 (0.3 %) consisting of nasal fractures, 4 (0.6 %) consisting of fractures of the atlantoaxial complex, and 1 (0.2 %) was a fracture of the orbital roof.

### 3.3 Patient outcomes

Five hundred twenty-seven (37.2 %) of the patients were institutionalized; this included 320 (41.5 %) from the At-risk group and 207 (32.2 %) from the Not-at-risk group (*p* < 0.001). Neurological deterioration, defined as a shift of 1 or greater in the mRS, was statistically more frequent in the At-risk group (31, 4.0 %) than in the Not-at-risk group (14, 2.2 %) (*p* = 0.050). Mortality during hospitalization was more frequent in the At-risk group (22, 2.8 %) than in the Not-at-risk group (11, 1.7 %), without reaching statistical significance. Traumatic injuries were the attributable cause of death in 2 (0.3 %) of cases for the At-risk group, and 0 (0.0 %) of cases for the Not-at-risk group .

Within the subset of patients who experienced intracranial hemorrhage, 6 (7.3 %) in the at-risk group and 0 (0.0 %) in the Not-at-risk group required neurosurgical intervention. A change in antithrombotic therapy was deemed necessary for 63 (76.8 %) of patients in the At-risk group and 3 (50.0 %) in the Not-at-risk group. A thrombotic event after change in antithrombotic therapy was observed in 1 (1.6 %) patient of the At-risk group and 0 (0.0 %) of the Not-at-risk group.

Table 3 summarizes characteristics and outcomes of patients of the Not-at-risk group who experienced intracranial hemorrhage.

**Table 3:** Study on the relevance of systematic head CT scans based solely on antithrombotic therapy in elderly patients aged 75 or older with mild traumatic brain injury: Characteristics and outcomes of patients of the Not-at-risk group who experienced intracranial hemorrhage.

Patient	Age	Gender	Therapy	Hemorrhage	Institutionalization	Neurosurgery	Neurological deterioration or death	Discontinuation of antithrombotic therapy	Thrombotic event
#1	80′s	Female	Aspirin	SAH	Yes	No	No	No	NA
#2	80′s	Male	VKA	IVH	No	No	No	Yes	No
#3	90′s	Female	VKA	SAH	No	No	No	Yes	No
#4	90′s	Female	Aspirin	SAH	No	No	No	No	NA
#5	90′s	Female	VKA	SAH	Yes	No	No	Yes	No
#6	90′s	Female	Aspirin	SDH	Yes	No	No	No	NA

VKA: vitamin K antagonist; SAH: subarachnoid hemorrhage; IVH: intraventricular hemorrhage; SDH: subdural hematoma; NA: not applicable.

## 4 Discussion

Recent literature indicates that conducting systematic head CT scans for mTBI in patients on antithrombotic therapy presents limited advantages, especially in adults under the age of 65 [1,3]. However, the necessity for such diagnostic procedures in older patients remains unclear. This study seeks to assess criteria that could potentially obviate the need for routine head CT scans, necessitated solely by the status of antithrombotic treatment, in elderly patients (75 years and older) presenting with mTBI. To the best of our knowledge, this represents the first investigation into this topic that specifically targets patients aged 75 and above, including a significant sample size: 1415 patients from January to December 2022.

Our study identified a traumatic hemorrhagic lesion rate of 6.2 % (*n* = 88/1415), aligning with the findings reported in the literature [3,14]. Among these patients, 6 (7.3 %) required neurosurgical intervention, and 2 (0.3 %) succumbed to their injuries. This raises questions about the necessity and efficacy of routine scanning based solely on the presence of antithrombotic treatment in elderly patients. However, considering the potential severity of hemorrhagic complications following head trauma, it is essential to identify predictive criteria for lesions beyond antithrombotic therapy alone. Doing so is vital for effectively selecting patients who genuinely need a scan.

In our study, patients were stratified into two groups based on criteria derived from current guidelines issued by France, Canada, the United Kingdom, and the United States [1,[11], [12], [13]]. The At-risk group consisted of patients presenting with any of the following conditions, either in isolation or combination: a GCS score of less than 15 or cognitive impairment; initial loss of consciousness; hemodynamic instability; evidence of skull vault, skull base, or facial fractures; extensive subcutaneous hematoma; severe or treatment-resistant headache; vomiting; seizure activity; any neurological deficit; drug or alcohol intoxication; amnesia concerning the event; or a history of neurosurgery. The Not-at-risk group included patients who did not meet any of these criteria. A statistically significant correlation was found between the presence of any of the aforementioned criteria and the occurrence of post-traumatic intracranial hemorrhage (*P* < 0.001), brain herniation (*P* = 0.003), or fractures (*P* < 0.001).

These results align with the study by Pages et al. [15] which revealed a low diagnostic and therapeutic yield of systematic scanning in patients older than 65 years following falls from their own height. It highlighted that abnormal GCS score, focal neurological deficit, and past history of post-traumatic brain injury were statistically associated with the presence of a lesion and should thus be considered in the decision-making process for imaging indication. Additionally, a study recently conducted by Dupuis et al. [2] aiming to evaluate the performance of a decision tree for indicating a brain CT scan in elderly patients with minor traumatic injuries, demonstrated that the majority (66.3 %) of hemorrhagic lesions requiring surgical intervention could be identified using these three criteria.

Only six post-traumatic hemorrhagic brain injuries were found in the group that did not present any of the studied criteria, and all these injuries were minor (localized SAH; millimetric SDH). Furthermore, none of these required immediate or delayed surgical intervention, and no neurological deterioration or deaths occurred in these patients. Regarding antithrombotic treatment, among the six patients in the Not-at-risk group who suffered a traumatic brain injury, none were receiving direct oral anticoagulants. Only patients on vitamin K antagonists (50 %, *n* = 3) had their antithrombotic treatment modified, and those taking aspirin (50 %, *n* = 3) underwent no therapeutic changes. The issue of discontinuing antithrombotic treatment in the event of minor intracranial bleeding following a mTBI remains uncertain. Indeed, the relevance of stopping a treatment with aspirin or direct oral anticoagulants is sometimes questionable, and the decision to continue these therapies often appears reasonable in light of the data available in the literature. Furthermore, even for vitamin K antagonists (VKAs), while it is widely accepted that discontinuation, possibly in conjunction with reversal, is necessary in the event of significant bleeding, it is not certain that they should be discontinued in the case of minor bleeding [[16], [17], [18], [19], [20], [21]]. Lastly, it's important to note that discontinuing antithrombotic treatment is not without consequences, as there is a low, but non-negligible, risk of thromboembolic complications [3]. In our study, a thrombotic event occurred in a patient following the cessation of antithrombotic therapy.

Seven fractures were observed in patients from Not-at-risk group, a group which theoretically does not present signs of fracture. This can be partly explained by the fact that our clinical examination only focused on fractures of the skull vault, base, or face, while four fractures of the atlanto-axial complex were identified through imaging. Additionally, nasal fractures are often difficult to date through imaging, and potentially old fractures may have been detected [22]. Lastly, the clinical examination is often challenging in these elderly and frequently agitated patients [23].

Due to the retrospective nature of this study and missing data, it was not possible to determine the precise average time interval between trauma and the completion of the CT scan. The concept of an optimal imaging delay is defined as the timeframe that minimizes the risk of morbidity and mortality associated with post-traumatic intracranial injuries. While early imaging may underestimate small hemorrhages or edema that are not yet visible, longer delays can postpone the diagnosis and treatment of lesions requiring urgent intervention. Performing CT scans as early as possible ensures the rapid detection of critical intracranial lesions and avoids delays that could worsen neurological outcomes. Delayed management of severe traumatic brain injuries has been shown to increase morbidity and mortality [24,25]. On the other hand, Geijerstam et al. [26] identified cases of neurological deterioration despite normal initial CT scans; however, these findings were based on a very limited number of cases and constrained by older imaging technologies and non-standardized methods. To address this, recent French national guidelines recommend performing CT scans as early as possible in patients with indications for urgent imaging. This aligns with other recommendations, such as the Canadian CT Head Rule [11] and the NICE guidelines [12], which demonstrate that timely imaging reliably identifies clinically significant lesions while ensuring optimal patient care.

The criteria used to categorize patients into the “At-risk group” and “Not-at-risk group” were inspired by widely recognized guidelines [1,[11], [12], [13]]. These guidelines aim to provide a comprehensive framework for identifying patients at higher risk of intracranial injury. While this approach appears effective in our study, its application in real-world clinical practice may present challenges. The number and complexity of the criteria require thorough and consistent assessment, which can be difficult to implement in high-pressure, time-sensitive environments like emergency departments. This emphasizes the need for further validation of these criteria in broader clinical settings and raises the question of whether a more streamlined set of criteria could achieve similar diagnostic accuracy while being more feasible in practice.

We acknowledge several limitations in our study. Firstly, being a single-center, observational, retrospective study, it may be subject to sampling bias, which could limit the generalizability of the results. Secondly, clinical data were retrospectively compiled from electronic patient records, sometimes necessitating the retroactive determination of the mRS and GCS scores from available information, rather than their direct calculation by emergency physicians during patient assessments. Thirdly, despite adherence to national guidelines, we cannot rule out potential selection bias due to the discretion exercised by emergency room physicians in deciding against CT scans for certain patients. Fourthly, we may not have captured some delayed intracranial hemorrhages due to the absence of a systematic follow-up protocol for patients post-discharge, although such occurrences are likely rare. Lastly, one notable limitation of our study is that our evaluation was based solely on clinical criteria; we did not investigate or utilize the blood assays for S100B, UCH-L1, or GFAP, which are of interest for limiting the number of head CT scans [1,4].

## 5 Conclusion

In conclusion, conducting systematic head CT scans based solely on antithrombotic therapy in elderly patients aged 75 or older with mTBI may be irrelevant. The criteria evaluated in this study suggest the possibility of postponing such CT scans. Applying these criteria could decrease the number of CT scans for this purpose by 45.4 %, representing a significant medical-economic saving. However, these results require validation through randomized controlled trials.

## Funding information

This research did not receive any specific grant from funding agencies in the public, commercial, or not-for-profit sectors.

## Ethical approval

All study protocols and procedures were conducted in accordance with the Declaration of Helsinki. According to French legislation, as a noninterventional retrospective study of routinely acquired data, the need for written informed consent for this study was waived.

## CRediT authorship contribution statement

**Emma Jaffres:** Formal analysis, Investigation, Resources. **Jean-Nicolas Dacher:** Validation, Writing – review & editing. **Mehdi Taalba:** Validation, Resources, Writing – review & editing. **Frédéric Roca:** Validation, Writing – review & editing. **Matthieu Garnier:** Validation, Writing – review & editing. **Sébastien Normant:** Validation, Writing – review & editing. **Mathieu Lozouet:** Validation, Writing – review & editing. **Emmanuel Gérardin:** Validation, Writing – review & editing. **Julien Burel:** Conceptualization, Methodology, Formal analysis, Investigation, Resources, Writing – original draft, Visualization, Supervision, Project administration.

## Declaration of competing interest

The authors declare that they have no known competing financial interests or personal relationships that could have appeared to influence the work reported in this paper.
